# Altitudinal and temporal distribution of *Plagiometriona* Spaeth, 1899 (Coleoptera, Chrysomelidae, Cassidinae) in a tropical forest in southeast Brazil

**DOI:** 10.3897/zookeys.157.1179

**Published:** 2011-12-21

**Authors:** Vivian Flinte, Sama de Freitas, Margarete Valverde de Macedo, Ricardo Ferreira Monteiro

**Affiliations:** 1 Laboratório de Ecologia de Insetos, Departamento de Ecologia, Instituto de Biologia, Universidade Federal do Rio de Janeiro. Rio de Janeiro, RJ, Brazil

**Keywords:** Species richness, abundance of individuals, population fluctuation, host plant, altitude, phenology, Chrysomelidae, Cassidinae, Solanaceae

## Abstract

Species richness and abundance of seven *Plagiometriona* species on their host plants were studied along a single trail in the mountainous Serra dos Órgãos National Park in the State of Rio de Janeiro, Brazil. Six sites were chosen along an altitudinal gradient ranging from 1300 m to 2050 m, where all Solanaceae host plants were inspected in search of adults every two months from June 2006 to June 2007. Species richness did not vary clearly with altitude, but abundance increased up to 1800 m, where the highest mean host plant density was found, and abruptly decreased at the last elevational site. Most species showed a restricted distribution and just one occurred across the entire gradient. For at least four species, altitudinal distribution seems to be strongly related to host plant availability, while for the others it is difficult to access which factors are decisive, due to their low numbers. Only in October all species were found in the field, although February was the month with the highest total abundance. Over the course of the study, the greatest abundances were recorded from October to February, comprehending the hottest and rainiest months, and the lowest abundances were found from June to August, which include the coldest and driest months. Thus, species seasonal distribution, supported by other studies in the same area, seems to be related to the local climate.

## Introduction

Chrysomelidae is one of the richest families of Coleoptera, comprised of almost 37,000 described species (Jolivet 1988). Species are almost exclusively phytophagous in habit and associated with host plants in a large number of Angiosperm families (Jolivet and Petitpierre 1976). The subfamily Cassidinae *s.l.*, i.e. including both “cassidoid” (tortoise beetles) and “hispoid” (hispines) forms (Staines 2002, Chaboo 2007, Bouchard et al. 2011) is the second largest subfamily within the chrysomelids, with 312 genera and approximately 6,000 species (Borowiec 1999, Staines 2004).

The majority of Cassidinae are specialized feeders (narrowly oligophagous), with few species which could be considered either truly monophagous or polyphagous in habits. In the neotropical region, cassidoid Cassidinae are mainly associated with host plants in the dicotyledenous families Convolvulaceae, Asteraceae, Bignoniaceae, Boraginaceae, Lamiaceae and Solanaceae. It is interesting to note how few potential plant families present in any particular area are actually exploited by these beetles. Of those plant families that are, most are members of a single clade of Eudicotyledonaes (Jolivet and Hawkeswood 1995, Soltis et al. 2011).

It is believed that multiple factors, operating across a hierarchy of spatial and temporal scales, shape species distributions (Levin 1992). Insect species distributions are influenced by abiotic factors (e.g. rainfall, humidity and temperature), biotic (e.g. host plants, predators/parasitoids), and by their physiology (Price 1975, Szukecki 1987). Recently, great focus is being given to the role of temperature due to the rising concern on how climatic change will affect species distribution (Bale et al. 2002, Battisti et al. 2006).

Nogueira-de-Sá et al. (2004) reviewed population phenology of Cassidinae *s.str.* in tropical and subtropical areas in Brazil and described different phenological patterns. Tropical cassidines tend to occur throughout the year and are little influenced by climatic factors and more influenced by host plant availability. In contrast, in subtropical areas the majority of species present a distinct period of reproduction and adults commonly overwinter in diapause. Reproduction of most of these species was observed only during the warmest and most humid seasons, as indicated by Wolda (1978, 1980).

On tropical mountains, abiotic factors are likely to have even greater effects on community structure, so patterns of population fluctuation similar to subtropical and even temperate regions may emerge, with occurrence periods of insects well defined throughout the year. Increasing altitude brings lower temperatures, increased precipitation (rain or snow), lower partial pressure of gases, higher wind speed and turbulence, and greater extremes in radiation input (Barry 1992). Combined, these factors may produce a general decrease in the structural complexity of insect habitats, as well as variation in the nutritional quality and availability of host plants. Phytophagous insects could well respond to these variations in host quality with changes in rates of growth, survival and fecundity (Hodkinson 2005).

Hodkinson (2005), in a review of terrestrial insects along elevational gradients, clearly shows that trends in species richness and abundance of individuals are variable, decreasing with increasing altitude (e.g. Wolda 1987, Fernandes and Lara 1993), increasing (e.g. Sota 1994, Romero and Avila 2000), peaking at middle elevation (e.g.Janzen 1973, Janzen et al. 1976, McCoy 1990), or showing no altitudinal trend (e.g. Casson and Hodkinson 1991). Many processes may explain species richness declines with increasing altitude, including reduced habitat area at high elevations, reduced resource diversity, increasingly unfavorable environments and reduced primary productivity (Lawton et al. 1987).

There are only few studies with Brazilian Chrysomelidae on elevational gradients, and data obtained so far show that abundance and richness patterns vary with altitude among study areas. Ribeiro et al. (1994) and Carneiro et al. (1995), working on the same gradient, found a tendency of increasing richness and abundance with increasing altitude and suggested that harsh climatic conditions on the mountain base might be responsible for this pattern. Flinte et al. (2009), studying chrysomelids on another gradient, did not record a clear variation of richness, but found a peak of abundance at an intermediate altitude. In this paper, we describe the altitudinal and temporal distribution of seven *Plagiometriona* Spaeth, 1899 (Cassidinae: Cassidini) species across the same elevational gradient in the State of Rio de Janeiro, Brazil, and focus on how elevational changes (specifically rainfall and temperature) and host plants can influence their distribution.

## Material and methods

### Study area

**Figure 1.**
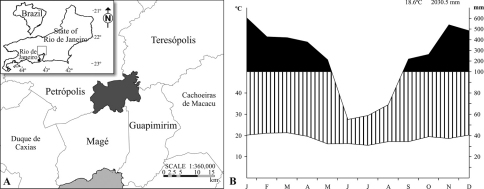
**A** Location of Serra dos Órgãos National Park in the State of Rio de Janeiro, Brazil and **B** climatic diagram (meteorological station at 980 m altitude, from the National Institute of Meteorology), compiled for the period of January 2007 to December 2008. Striped area= humid period; Black area= superhumid period.

Our study was conducted in Serra dos Órgãos National Park (22°32'S, 43°07'W), which encompasses an area of 20,024 ha, extending over four counties in southeast Brazil, State of Rio de Janeiro (Fig. 1A), within the tropical Atlantic domain. The climate is marked by mild summers, winters of high precipitation and temperature reduction with altitude (Castro 2008). The Park undergoes a superhumid period most of the year, marked by an intense rainfall, especially from November to March (mean of 458.2 mm monthly rainfall), while the drier season (though still humid) extends from June to August (mean of 48.8 mm monthly rainfall). The coldest months are between May and August (mean temperature of 16.4 °C), and the hottest fall in the period from December to March (mean temperature of 21.1 °C) (Flinte et al. 2009; Fig. 1B). Unfortunately, the meteorological station which provided the climatic data cited above was only installed in the Park in the middle of the present study, preventing proper correlation analysis with beetle richness and abundance. Four different types of vegetation, related to altitude, can be found in the Park (Rizzini 1954, Veloso et al. 1991): lower montane forest (below 800 m), montane (600–1500 m), high-montane (1500–2000 m) and high altitude grasslands, named *campos de altitude* (over 2000 m), characterized by shrubs, herbs and grasses.

Surveys were conducted at six sites of different altitudes (approximately 1300 m, 1500 m, 1600 m, 1700 m, 1800 m and 2050 m) in Teresópolis County, specifically on the *Pedra do Sino* trail, which has an altitudinal variation of more than 1000 m, ranging from 1100 m to 2263 m. Accordingly, the two lowest sites are found in montane forest, the highest occupies the high altitude grasslands, with intermediatesites in high-montane forest. Trail conditions (incident light, tree cover, humidity) were undoubtedly different from the interior of the forest, but these varied nevertheless along the chosen sites with changing phytophysiognomies. Besides, although host plant characteristics will probably not be the same, open areas such as clearings and trails may be preferable over intact canopy forest, since many Cassidinae, including various *Plagiometriona* species, are associated with secondary growth plants (Windsor, 1992). Flinte et al. (2009), studying Chrysomelidae along the same trail but at only three elevational sites, and with data from two meteorological stations at 980 m and 2140 m altitude, found lower rainfall volume at the latter, and a decrease of 0.61°C for each 100 m altitude, which means a difference of ca. 4.6°C between the lowest and highest sites of the present study.

### Study beetle species

**Figure 2.**
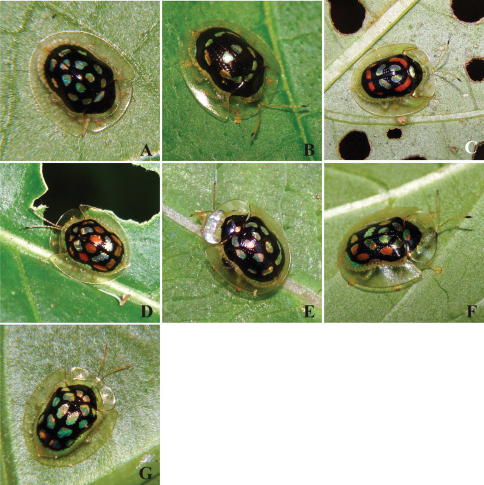
Species studied at the Serra dos Órgãos National Park: *Plagiometriona ambigena* **A** *Plagiometriona dodonea* **B** *Plagiometriona dorsosignata* **C** *Plagiometriona sahlbergi* **D** *Plagiometriona stillata* **E** *Plagiometriona tredecimguttata* **F** and *Plagiometriona* sp. 7**G**

Within a wider project on Chrysomelidae diversity and distribution started in 2005 in the Park, seven Cassidinae species were chosen for the present study: *Plagiometriona ambigena* (Boheman, 1855) (Fig. 2A), *Plagiometriona dodonea* (Boheman, 1855) (Fig. 2B), *Plagiometriona dorsosignata* (Boheman, 1855) (Fig. 2C), *Plagiometriona sahlbergi* (Boheman, 1855) (Fig. 2D), *Plagiometriona stillata* (Boheman, 1855) (Fig. 2E), *Plagiometriona tredecimguttata* (Boheman, 1862) (Fig. 2F) and *Plagiometriona* sp. 7 (Fig. 2G), all of which present very similar patterns of elytral coloration, form and body size. Within these species, the biggest individuals belong to *Plagiometriona dorsosignata* and the smallest to *Plagiometriona stillata* (mean of 69 and 52 mm, respectively; n= 10 each species). Some of these species (*Plagiometriona dodonea*, *Plagiometriona dorsosignata*, *Plagiometriona stillata* and *Plagiometriona tredecimguttata*) were also studied by Flinte et al. (2009) on the same altitudinal gradient, however using different methodologies.

To describe species richness on each plant species and altitude, only adults were considered because eggs, larvae and pupae of the studied *Plagiometriona* are very similar to each other and to other species of the same genus not mentioned here, but which can be found on the same plant species (this problem was already described by Flinte et al. 2009). Although immature stages are more sensitive to abiotic changes than adults, which limits our conclusions on the relative importance of such factors over beetle abundance, any factor affecting immatures would be reflected in adult abundance, not compromising the description of the temporal and spatial patterns considered here.

Adult individuals of the focal *Plagiometriona* species fed one or more of seven different Solanaceae species: *Aureliana fasciculata* Sendtn., *Capsicum mirabile* Mart., *Solanum campaniforme* Roem. & Schult., *Solanum enantiophyllanthum* Bitter, *Solanum megalochiton* Mart., *Solanum swartzianum* Roem. & Schult. (Solanoideae: Solaneae) and *Cestrum bracteatum* Link & Otto (Cestroideae: Cestreae) (Table 1).

**Table 1.** Host plant records based on larval “no choice” feeding tests for the seven *Plagiometriona* species in the study (from Flinte et al. 2008).

Host plants / Cassidines	*Aureliana fasciculata*	*Capsicum mirabile*	*Cestrum bracteatum*	*Solanum campaniforme*	*Solanum enantiophyllanthum*	*Solanum megalochiton*	*Solanum swartzianum*
*Plagiometriona ambigena*						x	x
*Plagiometriona dodonea*		x		x			
*Plagiometriona dorsosignata*	x						
*Plagiometriona sahlbergi*			x				
*Plagiometriona* sp. 7			x				
*Plagiometriona stillata*		x		x			
*Plagiometriona tredecimguttata*	x	x		x	x		

Because the host plants *Aureliana fasciculata* and *Solanum campaniforme* had very similar vegetative forms, it was not possible to reliably distinguish them in the field outside their reproductive season. Therefore, data from beetles associated with these plants were grouped in the present study.

Beetles were deposited in the collection of the Laboratório de Ecologia de Insetos at the Federal University of Rio de Janeiro, but some specimens were also deposited in the collection of the Department of Biodiversity and Evolutionary Taxonomy, Institute of Zoology, University of Wroclaw, Poland. After curation, plants were deposited in the Herbarium of the Federal University of Rio de Janeiro and in the Rio de Janeiro Botanical Garden.

### Temporal and altitudinal distribution

Surveys were conducted every two months from June 2006 to June 2007 by Sama de Freitas and one additional of four undergraduation students. At each site, two transects of 200 m × 0.5 m (length × width) were made, one at each side of the border of the trail. Within each transect, host plants were carefully surveyed for adults of the focal *Plagiometriona* species, and the number of individuals per plant and the number of each plant species was recorded in every site.

To obtain mean plant density for each altitude, we summed the number of plant individuals of each species in the transect per month, and divided that number by the number of surveys (seven months). In order to describe the temporal distribution of the species we considered the total abundance of each species per survey. Finally, beetle altitudinal distribution was calculated from the total number of adults sampled over the course of the study for each species and elevational site. Host plant quality was not considered in this study.

## Results and discussion

### Spatial distribution of host plants

**Figure 3.**
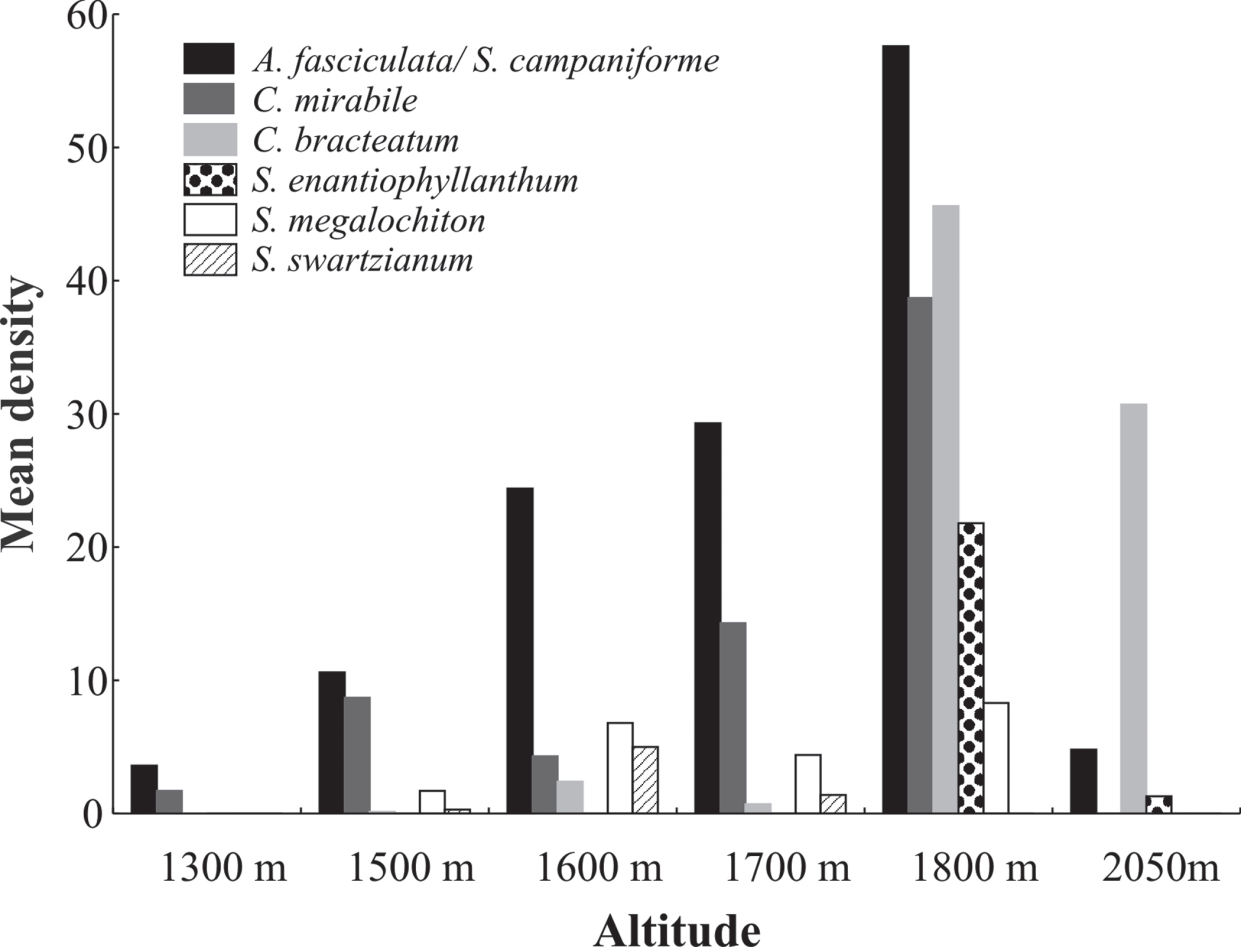
Mean host plant density of the studied *Plagiometriona* species at different altitudes.

At no altitudinal site did all seven host plant species co-occur. Plant richness was highest with five species co-occurring at 1600 m, 1700 m and 1800 m, and lowest at 1300 m and 2050 m where only two and three species co-occurred, respectively (Fig. 3). *Aureliana fasciculata* / *Solanum campaniforme* occurred along the whole elevational gradient, while the other species were more restricted in their altitudinal distribution. In general, host plant density was highest at 1800 m for all species, except *Solanum swartzianum*, which showed higher density at 1600 m.

### Occurrence of Plagiometriona spp. on their host plants

Our field observations were consistent with the data presented by Flinte et al. (2008) (Table 1), showing restricted feeding habits for the seven focal *Plagiometriona* species. *Plagiometriona dorsosignata*, *Plagiometriona sahlbergi*, *Plagiometriona stillata* and *Plagiometriona* sp.7 were associated with only a single host plant, while *Plagiometriona ambigena*, *Plagiometriona dodonea* and *Plagiometriona tredecimguttata* were locally oligophagous, having two or three related hosts (Table 2).

**Table 2.** Relative adult occurrence (in percentage) of the seven *Plagiometriona* species associated with Solanaceae at the study site.

Host plants / Cassidines	*Aureliana fasciculata* / *Solanum campaniforme*	*Capsicum mirabile*	*Capsicum bracteatum*	*Solanum enantiophyllanthum*	*Solanum megalochiton*	*Solanum swartzianum*
*Plagiometriona ambigena*					50.0	50.0
*Plagiometriona dodonea*	33.3	66.7				
*Plagiometriona dorsosignata*	100.0					
*Plagiometriona sahlbergi*	1.5*		98.5			
*Plagiometriona* sp. 7	3.7 *	3.7 *	92.6			
*Plagiometriona stillata*		100.0				
*Plagiometriona tredecimguttata*	70.9	25.5		2.9		0.7*

* Plant species on which adult individuals were found in field during the present study, but larval feeding was not recorded in the laboratory by Flinte et al. (2008).

Except for *Plagiometriona ambigena*, the other oligophagous species showed a clear preference for one of their host plants, with more than 50% of individuals being recorded on a single host species (Table 2). Because plants on the border of the trail are sometimes very close to each other, some individuals were eventually found on non-host plants, probably during dispersal or because of disturbances that may take place during the inspection of the feeding plants. Thus, very low percentage values of occurrence on “new” host plants were not considered true associations and must be confirmed by laboratory rearing. The occurrence of *Plagiometriona stillata* on only one of its described hosts in field may be an artifact due to the small number of individuals this species ever observed during the study period.

### Temporal and altitudinal distribution of Plagiometriona spp.

Abundance of the seven focal species varied considerably along the year. The lowest values were recorded in June, gradually increasing until peaking in February and then decreasing again (Table 3). Thus, the peak happens in the middle of the summer, when precipitation and temperatures are high, while the low numbers occur during months of lower rainfall and milder temperatures (Fig. 1B).

**Table 3.** Abundance (per month and total) of the seven *Plagiometriona* species studied from June 2006 to June 2007 at the study site, and species richness per month. Darker shade in gray indicates the month in which the most abundant species had the highest numbers of individuals.

Months / Cassidines	2006				2007			Total abundance
	**J**	**A**	**O**	**D**	**F**	**A**	**J**	
*Plagiometriona ambigena*	0	**1**	**1**	0	**2**	0	0	**4**
*Plagiometriona dodonea*	0	0	**3**	0	0	0	0	**3**
*Plagiometriona dorsosignata*	**4**	**16**	**6**	**15**	**24**	**3**	**2**	**70**
*Plagiometriona sahlbergi*	**3**	**15**	**30**	**22**	**58**	**9**	0	**137**
*Plagiometriona* sp. 7	0	**2**	**1**	**3**	**19**	0	**2**	**27**
*Plagiometriona stillata*	0	0	**1**	0	0	0	0	**1**
*Plagiometriona tredecimguttata*	**4**	**10**	**26**	**37**	**39**	**18**	**7**	**141**
**Total abundance**	**11**	**44**	**68**	**77**	**142**	**30**	**11**	**363**
**Species richness**	**3**	**5**	**7**	**4**	**5**	**3**	**3**	

In spite of the low number of studied species, changes in species richness could also be observed during the period; in June 2006 and 2007 and April 2007 just three of the four most abundant species were found in the field and only in October all seven species were recorded together (Table 3). Once again, the lowest numbers occur in the coldest and driest months, and the highest during the rainy season with warmer temperatures.

Three species, *Plagiometriona dorsosignata*, *Plagiometriona sahlbergi* and *Plagiometriona tredecimguttata* were the most abundant species during the study. Also, *Plagiometriona dorsosignata* and *Plagiometriona tredecimguttata* were the only two species present in all surveyed months. Although *Plagiometriona* sp. 7 occurred practically throughout the year, the total number of individuals recorded was very small, with the exception of the February survey when its abundance was the highest observed. *Plagiometriona ambigena*, *Plagiometriona dodonea* and *Plagiometriona stillata* were rarely observed over the entire study (Table 3). The two latter were only recorded once and in October.

**Figure 4.**
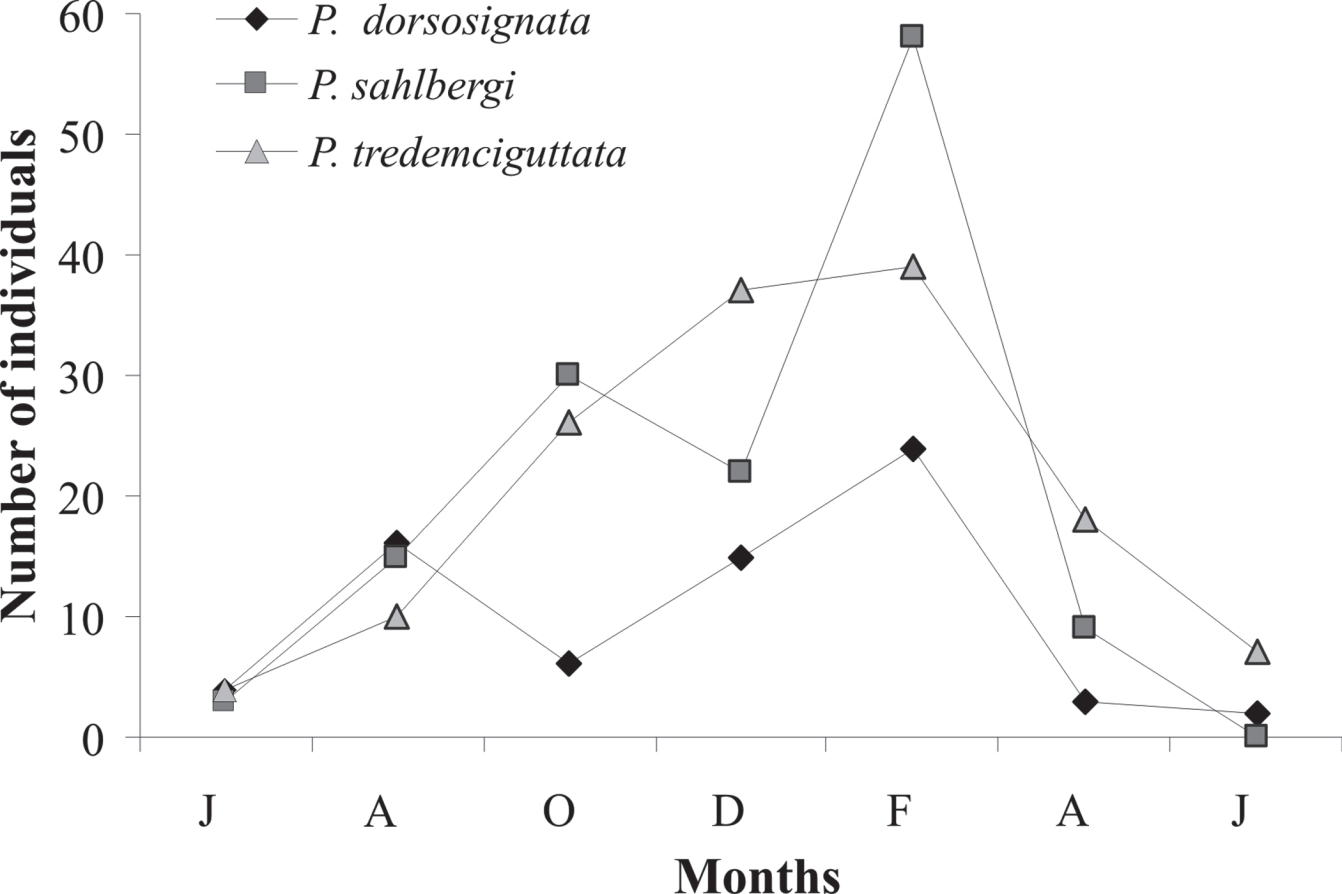
Population fluctuation of *Plagiometriona dorsosignata*, *Plagiometriona sahlbergi* and *Plagiometriona tredecimguttata* from June 2006 to June 2007 at the study site.

*Plagiometriona dorsosignata*, *Plagiometriona sahlbergi*, and *Plagiometriona tredecimguttata* numbers varied similarly during the study period. Their abundance was very low in June 2006, peaked in February 2007 and decreased again in April (Fig. 4). Increasing numbers during the study may reflect temperature and precipitation increases, since the highest numbers of individuals were found from October to February, comprehending the warmest and most rainy months, while the lowest were recorded from June to August, the coldest and driest period (Fig. 1B). This increase in cassidine activity during the warm and rainy season was also described by Nogueira-de-Sá et al. (2004), in a review of the subfamily’s phenology in Brazil. As seasonal changes in temperature are very slight in the tropics, it is believed that the seasonal distribution of rainfall exerts a greater influence on insect population dynamics (Delinger 1986, Wolda 1988). However, on an altitudinal gradient, temperature variation has a decisive influence even in the tropics, as every 1000 m altitude results in a decrease in temperature of about 6°C (Odgen and Powell 1979).

Most insect species living in temperate zones become active during spring and summer, overwintering in diapause (Wolda 1988). In the tropics, even though seasons are not as well defined, some beetle species also show activity peaks, as the chrysomeline *Platyphora anastomozans* (Medeiros and Vasconcellos-Neto 1994), which is more abundant between October and May, overwintering in diapause. An activity peak was also recorded for the three most abundant species of our study, *Plagiometriona dorsosignata*, *Plagiometriona sahlbergi* and *Plagiometriona tredecimguttata*, although no evidence of diapause was found, at least for adults.

Changes in community composition per elevational site were observed with increasing altitude. Considering the small number of study species, richness showed no clear pattern with altitude, starting with three species at the three lowest sites, to four species at intermediate elevations (1700 m and 1800 m), and decreasing to two species at the highest site (Table 4).

**Table 4.** Abundance of the seven *Plagiometriona* species at the different altitudinal sites, number of species per altitude and total of sites in which each species was recorded at the study area. Darker shade in gray indicates the altitudinal sites in which the most abundant species had the highest numbers of individuals.

Altitudes / Cassidines	1300 m	1500 m	1600 m	1700 m	1800 m	2050 m	Total abundance	Total sites
*Plagiometriona ambigena*	0	0	**3**	**1**	0	0	**4**	**2**
*Plagiometriona dodonea*	**2**	**1**	0	0	0	0	**3**	**2**
*Plagiometriona dorsosignata*	0	**2**	0	**41**	**27**	0	**70**	**3**
*Plagiometriona sahlbergi*	0	0	**5**	**4**	**96**	**32**	**137**	**4**
*Plagiometriona* sp. 7	0	0	0	0	**27**	0	**27**	**1**
*Plagiometriona stillata*	**1**	0	0	0	0	0	**1**	**1**
*Plagiometriona tredecimguttata*	**1**	**14**	**7**	**13**	**103**	**3**	**141**	**6**
**Total abundance**	**4**	**17**	**15**	**61**	**252**	**35**	**383**	
**Species richness**	**3**	**3**	**3**	**4**	**4**	**2**		

Significant differences occurred in the spatial distribution of *Plagiometriona* species along the altitudinal gradient; with some species, such as *Plagiometriona* sp. 7 and *Plagiometriona stillata* restricted to a single altitude, while others showed a wide elevational distribution, *Plagiometriona tredecimguttata* being the most remarkable example, occurring on all six elevational sites. Most species were restricted in their altitudinal range, occurring at two, three or four sites, normally adjacent to each other (Table 4).

A general increase in the total abundance of beetles throughout the altitudinal gradient was recorded, the highest numbers of individuals being found at 1700 m and 1800 m, 61 and 252, respectively, followed by a sharp decrease in abundance at the highest site (Table 4). This pattern was evident in the distributions of each of the three most numerous species.

*Plagiometriona* sp. 7 and *Plagiometriona sahlbergi* feed on the same host plant, *Cestrum bracteatum*, which was observed from 1600 to 2050 m. The highest density of *Plagiometriona bracteatum* occurred at 1800 m (Fig. 5A), where 57.3% (n= 557) of all its individuals were recorded. At this site we also observed the highest abundance of *Plagiometriona sahlbergi*, which was found at the same altitudinal range as its host plant (Fig. 5A). Thus, spatial distribution of *Plagiometriona sahlbergi* along the elevational gradient seems to be greatly influenced by the availability of its host plant. Individuals of *Plagiometriona* sp. 7 were only found at 1800 m (Fig. 5B), suggesting that their occurrence may be responding to the high density of their host at this altitude. Meanwhile, at the highest elevational site, where plant density is still considerable, the species absence may be explained by the lack of physiological adaptations necessary to survive at such altitudes. With increasing altitude, abiotic factors such as temperature and precipitation can influence physiological and morphological changes in insect populations in the short term (e.g. variations in the life cycle, fecundity and size of individuals) and over evolutionary time (e.g. such as high numbers of apterous or brachypterous individuals, polymorphisms) (Hodkinson 2005, Chown and Klok 2003).

**Figure:**
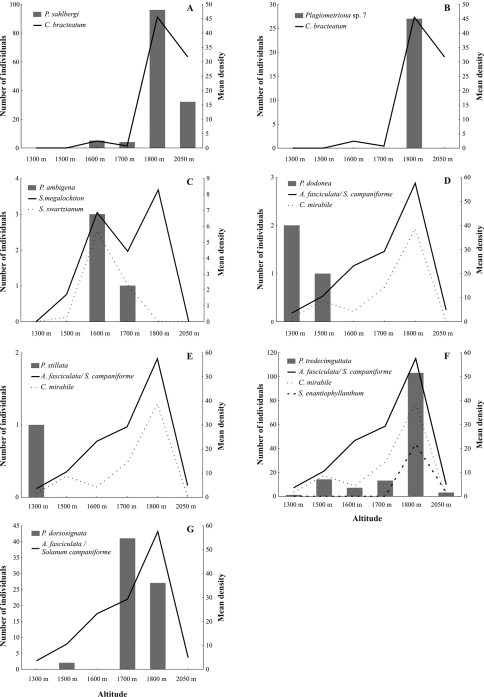
**Figure 5.** Altitudinal distribution of the seven *Plagiometriona* species (bars) and their host plants (lines) from June 2006 to June 2007 at the study site: *Plagiometriona sahlbergi* **A** *Plagiometriona* sp. 7 **B** *Plagiometriona ambigena* **C** *Plagiometriona dodonea* **D** *Plagiometriona stillata* **E** *Plagiometriona tredecimguttata* **F** and *Plagiometriona dorsosignata* .

*Plagiometriona ambigena*, *Plagiometriona dodonea* and *Plagiometriona stillata* were restricted to a small altitudinal range, occurring at only one or two sites, while their host plants showed broader distribution along the elevational gradient (Fig. 5C, D and E, respectively). Thus, it is possible that these three species also have low tolerance to the harsher climatic conditions at higher sites, which prevent them from having a wider altitudinal range. Another possibility may be sampling error, in that their small numbers prevented us from recording them at different altitudes.

*Plagiometriona tredecimguttata* occurred throughout the entire elevational gradient of the study (Fig. 5F), but not in even numbers. Low abundances were recorded at the lowest and highest sites of the gradient, with numbers peaking at 1800 m. In this way, species density increased with altitude to 1800 m, after which it decreased, following variation in host plant density (Fig. 5F). Therefore, the distribution of *Plagiometriona tredecimguttata* appears strongly related to host availability.

*Plagiometriona dorsosignata* was found from 1500 m to 1800 m, with its abundance peaking at 1700 m (Fig. 5G). Its host plant *Aureliana fasciculata* is potentially distributed along the entire length of the transect, but more densely between 1700 m and 1800 m, where the beetle is also more abundant.

A general pattern observed within the seven study species seems to be the complete or near absence of most of the species at the highest altitudinal site. Temperatures below 0°C are commonly recorded at the highest elevations in the Park (Castro 2008), demanding morphological and, specially, physiological adaptations to enable beetle survival (Gaston and Chown 1999). Climatic factors, in particular temperature, may thus influence beetles directly or indirectly via host plant. Although host plant quality was not analyzed here, plant availability appears to be a decisive factor, clearly influencing the altitudinal distribution of four out of the seven chrysomelids studied. However, even in the case of the other three species, host plant influence cannot be discarded, since the number of these beetles was very low. In that way, differences in numbers of individuals and species composition along the altitudinal gradient may depend on host plant availability, but also on species coping differently with varying abiotic conditions related to altitude. Naturally, the role of competing species, predators and parasitoids cannot be ruled out as another force determining the distribution of the species. According to Hodkinson (2005), knowledge of tritrophic interaction between host plant, herbivorous insect and predators/parasites, although rare, may improve the understanding of population dynamics along altitudinal gradients. Unfortunately, we are only beginning our research on insect distribution along such gradients in the tropics and much work still remains to clarify the factors underlying species distribution.

Flinte et al. (2009), studying 12 Chrysomelidae species, being eight cassidines (including *Plagiometriona dorsosignata*, *Plagiometriona dodonea*, *Plagiometriona stillata* and *Plagiometriona tredecimguttata*), along the same trail one year later, found that species richness did not vary clearly with altitude, but recorded a distinct abundance peak at mid-elevational sites (1600-1800 m). Furthermore, both species richness and abundance showed a drastic reduction during the driest and coldest months, and high numbers when temperature and rainfall increased. Flinte et al. (2010) also described the altitudinal and seasonal pattern in abundance of another related Cassidinae in the same gradient, *Plagiometriona forcipata* (= *Plagiometriona emarcida*)which feeds on *Solanum lhotskyanum*. Their findings support the well defined seasonal distribution already known for the area, however, both adults and larvae showed higher numbers of individuals at the high- (2000-2100 m) than at the mid-elevation site (1600-1800 m). Although dealing with another species on a different host plant, this suggests that immature stages may respond similarly as adults to changing factors related to altitude.

Since many Cassidinae are associated with host plants on open habitats (Windsor 1992), surveys conducted on trails with a genera composed by many sun-loving species, such as *Plagiometriona*, may be a good indicator of the spatial and temporal distribution of the group. There is no doubt that descriptive studies such as the one presented here are an important starting point to widen our knowledge on cassidine ecology.
